# 

**Published:** 1904-10

**Authors:** 


					﻿CONTENTS.
ORIGINAL COMMUNICATIONS—
■
Cataract Operations—Report of One Hundred and Three Cases. By T. M. McIntosh, iM.D.,
Thomasville, Ga....................................................... 433
Address to Graduating Class of Grady Hospital, 1904. By C. D. Hurt, M.D , Atlanta, Ga. 444
Individual Peculiarities of the Male Urethra. By W. B. Emery UM.D , Atlanta, Ga....... 449
EDITORIAL NOTES AND COMMENTS—
Rape and Its Penalty................................................................... 4K
Medical Commission on Tuberculosis for the state of Georgia............................ 4S
NEWS AND NOTES............................................................   4.1
CONTENTS CONTINUED............................................................ fl
CONTENTS—CONTINUED.
BOOK REVIEWS—
Books Received..............................................•....... 460
Lea’s Series of Medical Epitomes.................................... 460
SELECTIONS AND ABSTRACTS—
Typhoid Fever....................................................... 461
The Abortive Treatment of Gonorrhea in the Male..................... 467
Constipation........................................................ 474
Success in Medicine, and the Way It May Be Achieved................. 478
Hysteria and Crime Mimicry.......................................... 484
Delirium............................................................ 488
Sundown Journalism.................................................. 492
Shall We Amend Our Therapeutics and Our Prognosis?.................. 497
Dietetic Nuggets.................................................... 499
The Dangers of Acetanilid .......................................... 501
The Training of Patients............................................ 502
MEDICAL ITEMS—Continued......................xvi, xviii, xx, xxii, xxiv, xxvi, xxxii.
				

